# P28 Antibiotic susceptibility testing of coagulase-negative staphylococci species isolates from the Royal Orthopaedic Hospital, Birmingham

**DOI:** 10.1093/jacamr/dlaf118.035

**Published:** 2025-07-14

**Authors:** Uzair Akbar Ali, Saad Mahmood, Gareth Hughes

**Affiliations:** University Hospitals Birmingham NHS Foundation Trust, Birmingham, UK; University Hospitals Birmingham NHS Foundation Trust, Birmingham, UK; University Hospitals Birmingham NHS Foundation Trust, Birmingham, UK

## Abstract

**Background:**

Coagulase-negative staphylococci (CNS) are often considered non-pathogenic; however, they can be a major cause of chronic prosthetic joint infections (PJIs), associated with significant morbidity, mortality, and healthcare costs in the UK. Oral antibiotic options are often limited due to resistance, making IV glycopeptide antibiotics (vancomycin and teicoplanin) the preferred first-line treatment. While vancomycin-resistant isolates are rare, susceptibility to teicoplanin can vary, with some studies indicating that resistance may occur in up to a quarter of cases. Teicoplanin once daily administration provides significant benefits for managing outpatient treatment of certain patients with PJIs, compared to vancomycin, which requires twice-daily dosing.

**Objectives:**

The study aimed to assess local antibiotic susceptibility data in CNS over the past five years, particularly in patients with bone and joint infections from the Royal Orthopaedic Hospital, Birmingham. The secondary objective was to determine the teicoplanin, daptomycin, and linezolid susceptibility profile of the included isolates.

**Methods:**

This retrospective study involved a search of the Laboratory Information Management System (Telepath) to identify all isolates of CNS, including *Staphylococcus epidermidis*, *Staphylococcus haemolyticus*, *Staphylococcus hominis* and *Staphylococcus capitis* from bone, tissue, or fluid samples obtained from patients who underwent operations at the Royal Orthopaedic Hospital in Birmingham, one of the largest centres of its kind in Europe. MALDI-TOF MS (bioMérieux) was used for rapid and accurate species identification, while VITEK2^®^ (bioMérieux) was employed for antimicrobial susceptibility testing. Teicoplanin susceptibility was assessed using the Etest^®^ teicoplanin (bioMérieux) and EUCAST breakpoints (≤4 = susceptible, >4 = resistant).

**Results:**

We identified a total of 683 specimens that met the eligibility criteria. After excluding duplicate isolates and focusing on those that underwent a teicoplanin e-test, we included 47 isolates in the study. The median age of the patients was 69 years, including 29 males (62%) and 18 females (38%). The primary infection sites included the knee joint (36%) and hip joints (34%), followed by the shoulder, ankle joints, and miscellaneous specimens. *S. epidermidis* was the predominant species isolated from the analysed specimens (*n*=45, 96%). All isolates showed susceptibility to vancomycin, daptomycin, and linezolid. The highest antibiotic resistance recorded was for clindamycin (*n*=39, 83%), followed by trimethoprim (*n*=37, 79%) and fluoroquinolones (ciprofloxacin, 65%). Susceptibility testing for additional oral agents revealed significant resistance to tetracycline (*n*=28, 60%). and rifampicin (*n*=20, 42%). Results for teicoplanin susceptibility indicated that 16 (34%) isolates exhibited resistance based on EUCAST breakpoints. Among all isolates, 21 (45%) were sensitive to at least one oral option besides linezolid. Notably, we observed an increased rate of teicoplanin resistance in 2023 and 2024 compared to 2020 and 2022. The study did not assess the impact of antibiotic use or other factors that could contribute to antibiotic resistance.

**Conclusions:**

*S. epidermidis* was the predominant species identified among CNS associated with bone and joint infections. Most isolates exhibited resistance to commonly prescribed oral antibiotics. Furthermore, there has been a growing resistance to teicoplanin over the years, necessitating the need for further studies to explore the underlying factors.

